# Sex, drugs and gender roles: mapping the use of sex and gender based analysis in pharmaceutical policy research

**DOI:** 10.1186/1475-9276-9-26

**Published:** 2010-11-19

**Authors:** Devon L Greyson, Annelies RE Becu, Steven G Morgan

**Affiliations:** 1 Centre for Health Services and Policy Research, School of Population and Public Health, University of British Columbia, 201-2206 East Mall, Vancouver, BC, V6T1Z3, Canada

## Abstract

**Background:**

Sex and gender sensitive inquiry is critical in pharmaceutical policy due to the sector's historical connection with women's health issues and due to the confluence of biological, social, political, and economic factors that shape the development, promotion, use, and effects of medicinal treatments. A growing number of research bodies internationally have issued laws, guidance or encouragement to support conducting sex and gender based analysis (SGBA) in all health related research.

**Methods:**

In order to investigate the degree to which attempts to mainstream SGBA have translated into actual research practices in the field of pharmaceutical policy, we employed methods of literature scoping and mapping. A random sample of English-language pharmaceutical policy research articles published in 2008 and indexed in MEDLINE was analysed according to: 1) use of sex and gender related language, 2) application of sex and gender related concepts, and 3) level of SGBA employed.

**Results:**

Two thirds of the articles (67%) in our sample made no mention of sex or gender. Similarly, 69% did not contain any sex or gender related content whatsoever. Of those that did contain some sex or gender content, the majority focused on sex. Only 2 of the 85 pharmaceutical policy articles reviewed for this study were primarily focused on sex or gender issues; both of these were review articles. Eighty-one percent of the articles in our study contained no SGBA, functioning instead at a sex-blind or gender-neutral level, even though the majority of these (86%) were focused on topics with sex or gender aspects.

**Conclusions:**

Despite pharmaceutical policy's long entwinement with issues of sex and gender, and the emergence of international guidelines for the inclusion of SGBA in health research, the community of pharmaceutical policy researchers has not internalized, or "mainstreamed," the practice. Increased application of SGBA is, in most cases, not only appropriate for the topics under investigation, but well within the reach of today's pharmaceutical policy researchers.

## Background

Health researchers are increasingly investigating the ways that social and biological factors interact as determinants of health. The influences of biological and social dimensions of sex and gender are important in this regard. Although medical research has historically focused on issues related to sex (the biological attributes linked to the categories of male and female) rather than gender (the social constructs culturally linked to "maleness" and "femaleness") [1,2], a growing body of research suggests that health status, access to care, and medical outcomes are influenced by an individual's status in society, including one's status as a sexed and gendered being [3]. Similar to race and ethnicity studies, high-quality sex and gender based analysis (SGBA) can help document inequities in health and health care, advance understanding of needs, and improve population and individual health outcomes. This potential for a more sophisticated understanding of health needs and outcomes within and among identified demographic groups - such as men and women - is greater still when researchers apply intersectional analysis techniques, examining how various social categories combine and interact to create difference.

SGBA may be defined as, "an approach to research and evaluation which systematically inquires about biological (sex-based) and sociocultural (gender-based) differences between women and men, boys and girls, without presuming that any differences exist" [4]. Sex and gender sensitive inquiry is particularly critical in areas of pharmaceutical policy due to the sector's historical connection with women's health issues and due to the confluence of biological, social, political, and economic factors that shape the development, promotion, use, and effects of medicinal treatments [4,5]. The women's health movement has successfully advocated for more research on women's health [6,7], and particularly for women's inclusion in clinical trials [8,9], resulting in 1993 United States legislation [10] and 1997 Canadian guidelines [11] on inclusion of women in drug trials. A growing number of research bodies internationally have also issued guidance and encouragement to support conducting "gender and sex-based analysis" [12] or "gender mainstreaming" [13] in all health related research.

While the value of high-quality SGBA in pharmaceuticals has been established, it is as yet unclear whether SGBA has permeated the field of pharmaceutical policy research. Have the attempts to mainstream SGBA translated into sex and gender sensitive research practices in the field of pharmaceutical policy? In order to address this question, we undertook a literature scoping exercise to map the extent, range and nature of current practices related to SGBA in a representative sample of English language pharmaceutical policy research.

## Methods

We employed methods of literature scoping and mapping [14-16], beginning with a search of the MEDLINE database (daily update, 1950-present, via Ovid SP interface) conducted by DG on July 16, 2009 for English language articles on pharmaceuticals and public policy that were published in 2008 (see Appendix 1 for search strategy). Abstracts of potentially relevant citations were screened by DG for pharmaceutical policy relevance, defining pharmaceutical policy as, "the rules, processes, and structures that are put in place by governments and public agencies to manage problems related to the availability of medicines and the role of medicine in health care"[17]. We excluded articles not directly related to pharmaceutical policy (such as those on illicit drug policy or environmental health) and non-policy articles on pharmaceuticals (such as clinical studies of a particular drug), as well as articles not containing original research or review content. After screening citations, we drew a random sample of articles for detailed analysis and classification.

DG and AB coded the sample of included articles using a standardized extraction and classification template. This template was developed through an iterative process in which themes from the literature on SGBA and pharmaceuticals were transformed into coding categories applicable to a wide range of pharmaceutical policy research, and pilot tested on a separate sample of articles from the core journal *Health Policy *in order to achieve >90% inter-rater reliability before proceeding through the included studies. Disagreements in coding of included studies were reconciled via discussion between the coders. The template allowed for categorical coding of each article's research methods and data sources, binary coding of details about whether and how sex and/or gender was used in both the language and concepts applied in the article, and categorical coding of the level of SGBA employed in the article. In addition, the template provided a free text section to capture additional aspects of interest - ranging from contextual information that might lend insight into the SGBA choices of the authors (for example, special attributes of the data or population studied), to notes on ways SGBA might have been, but was not, included.

We coded articles' sex or gender *language *in terms of the use of words indicating sex or gender in the text of the article, for example a statement that " male survey respondents were more likely to strongly agree " or "46% of the study sample were women." We also coded for language that was related to, but not synonymous with, sex and gender, such as sexual orientation and pregnancy status. We coded for sex or gender *application *referring to use of sex or gender concepts in the underpinnings or research analysis of an article; for example a statistical analysis using sex disaggregated data, or the consideration of gender norms in a policy analysis. The intent of this coding was to assess the extent to which any sex or gender language and concept use was taking place in our sample. While language and content of an article are related, it is possible for an article to make mention of sex or gender yet not apply it in any way conceptually or analytically, or to use language that does not accurately represent the concepts being applied or investigated; therefore examination of both among articles in our sample was necessary.

Beyond assessing language and conceptual inclusion of sex and gender, we assessed whether the studies in our sample replicated themes commonly identified in the literature as challenges to achieving "mainstreamed" gender and sex analyses. We coded whether "sex" or "gender" were defined, and whether sex and gender were used interchangeably or otherwise conflated. For articles using quantitative research methods, we coded whether sex and/or gender was a variable in analysis, whether the analysis was sex disaggregated, and - if studying a phenomenon or condition that affected women as well as men - whether at least 45% of the population studied was female. For qualitative studies, we coded whether or not sex and/or gender were considered by the study authors as a theme of the analysis.

Finally, adapting Varcoe, Hankivsky, and Morrow's hierarchy of "Four approaches to thinking about women's health" [18], we coded articles as to the level of SGBA at which they functioned. Level 1 is the gender-neutral or sex-blind approach, in which sex and gender are ignored or seen as irrelevant to health. Level 2 comprises approaches reliant on biological essentialism or determinism. Level 3 is the level at which sex and/or gender based analysis is applied, and sex and/or gender are viewed as significant determinants of health beyond reproductive and sexual capacity alone. Level 4 includes research in which sex and gender are incorporated into intersectional analysis, which views sex and gender as interdependent with other social determinants, such as socio-economic status, culture, sexual orientation, age, and dis/ability. We flagged Level 1 articles for which SGBA could reasonably be excluded based on the study subject, for example economic studies of competition between firms or policy analyses about non-gendered phenomena. Studies with human data and studies of drug or policy impacts on humans were ineligible to be considered to appropriately function in a gender-neutral and sex-blind manner.

## Results

### Articles

**Figure 1:**
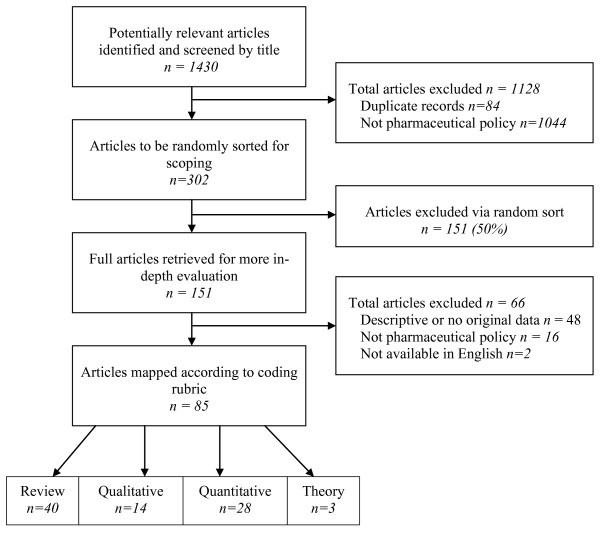
**Search and screening process for literature mapping**.

Our original search of the MEDLINE database produced 1,346 unique citations published in 2008 and potentially related to pharmaceuticals and public policy. Title and abstract reviews reduced the list of potential articles for our study to 302. We drew a random sample of 151 of these (50%) for full text analysis. Upon full text analysis, 66 further articles were excluded on the basis of being off-topic, not including original research, or being unavailable in English. The remaining 85 original research articles about pharmaceutical policy were subject to full data extraction and classification; 14 of these articles used qualitative research methodologies, 28 used quantitative methods, 40 were review articles, and 3 were theory pieces. Figure 1 illustrates the search and screening process.

### Use of sex and gender language

Table 1 summarizes aspects of sex and gender language and application in the 85 articles included in our study. Fifty-seven of the articles (67% of the total) made no mention of sex or gender. Of those containing some sex or gender language, 11 articles (13%) mentioned sex, 8 articles (9%) mentioned gender, and 21 articles (25%) used language that was sex or gender related but not clearly one or the other. Of the 21 studies using sex and/or gender language that was ambiguous, the potentially related language focused on concepts such as pregnancy, hormones, transsexuals, or women, without clarity on whether the concepts were intended to be understood in terms of the biology of sex, social dimensions of gender, or both. None of the papers in our sample offered a definition of sex or gender, and 7 articles (8%) used the words sex and gender interchangeably or otherwise conflated their meaning.

**Table 1:** Sex and gender language and application by research type among articles included in study

	Qualitative articles (n = 14)	Quantitative articles (n = 28)	Review articles (n = 40)	Theory articles (n = 3)	Total (n = 85)
**Sex/gender conflation**	0% (0)	7% (2)	10% (4)	33% (1)	**8% (7)**

**Language**					

Gender	7% (1)	7% (2)	10% (4)	33% (1)	**9% (8)**

Sex	0% (0)	14% (4)	18% (7)	0% (0)	**13% (11)**

Related/Ambiguous*	36% (5)	11% (3)	30% (12)	33% (1)	**25% (21)**

None	64% (9)	75% (21)	63% (25)	67% (2)	**67% (57)**

**Application**					

Gender	7% (1)	0% (0)	10% (4)	33% (1)	**7% (6)**

Sex	21% (3)	21% (6)	20% (8)	33% (1)	**21% (18)**

Related/Ambiguous**	14% (2)	4% (1)	13% (5)	33% (1)	**11% (9)**

None	64% (9)	75% (21)	68% (27)	67% (2)	**69% (59)**

* Related/Ambiguous language focused on concepts such as women, pregnancy, hormones, and transsexuals.

** Related/Ambiguous application included such concepts as: pregnancy, sex hormones, gender identity, sexuality, and sexual orientation

### Application of sex and gender concepts

Fifty-nine of the articles in our sample (69% of the total) contained no sex or gender related content whatsoever, not even a note that sex or gender data was unavailable for that study. This number is slightly higher than the number without sex or gender language, as two of these articles had used sex or gender language somewhere in the article but did not actually apply any sex or gender concepts in any part of the article. Of those containing some level of sex or gender concept application, 18 articles (21%) included some form of sex-based content, 6 articles (7%) included some form of gender-based content, and 9 (11%) incorporated related concepts. Some of these articles applied SGBA to the research that was the primary focus of the article, whereas others only included it as part of the article's conceptual background or literature review section. Methods and approaches to applying sex and gender concepts varied widely within and across study types.

SGBA was the main focus of none of the 5 qualitative papers [19-23] that considered some aspect of sex, gender or related concepts. Of the 6 quantitative articles that included some degree of sex content, just 1 [24] included sex-disaggregated analysis; 3 others [25-27] used sex as a variable for adjusting/controlling for sex-related effects (often unspecified). Only 3 [24,25,27] of all 28 quantitative articles clearly included study populations that were known to be >45% female, although several either did not know or did not disclose the sex breakdown of human populations studied, so this issue is difficult to assess in our sample.

Only 2 of the 85 pharmaceutical policy articles reviewed for this study were primarily focused on sex or gender issues. Both of these were review articles: one on the topic of transsexuality treatment options and another on medical abortion drug approval [28,29]. A third review article [30], focusing on clinical trials policy, contained a significant focus on sex and gender issues. All other sex or gender analyses were minor portions of the main articles.

### Levels of SGBA

As illustrated in Table 2, 69 (81%) of the studies included in our analysis conducted research at Level 1, a gender-neutral or sex-blind approach. Fifty of these articles (59% of all included studies) took such an approach even when focusing on topics with possible sex or gender aspects. Quantitative research articles were most likely to be gender-neutral or sex-blind; however, a higher proportion of the qualitative studies that took a Level 1 approach did so when SGBA could reasonably be expected of the subject matter (79% of Level 1 qualitative studies vs. 75% Level 1 quantitative studies).

**Table 2:** Levels of SGBA found among articles in study sample

	Level 4: Inter-sectional	Level 3: SGBA applied	Level 2: Biological essentialism/ determinism	Level 1: Gender-neutral or sex-blind	Level 1: SGBA reasonably excluded	Level 1: SGBA reasonably expected
**Qualitative articles**	0 (0%)	0 (0%)	3 (21%)	11 (79%)	0 (0%)	11 (79%)

**Quantitative articles**	0 (0%)	1 (4%)	1 (4%)	26 (93%)	5 (18%)	21 (75%)

**Review articles**	0 (0%)	4 (10%)	6 (15%)	30 (75%)	13 (33%)	17 (43%)

**Theory articles**	0 (0%)	1 (33%)	0 (0%)	2 (67%)	1 (33%)	1 (33%)

**Total**	0 (0%)	6 (7%)	10 (12%)	69 (81%)	19 (22%)	50 (59%)

Ten of our study articles (12%) took a Level 2 approach, either assuming sex and gender by virtue of focusing on a sexual or reproductive-related topic, or by equating men or women's health with gendered body parts, conditions or therapies (e.g., breasts, pregnancy, or sildenafil). Six of our study articles (7%) took a Level 3 approach, employing some sort of SGBA in the work reported. None of the articles employing qualitative research methods - the methods with perhaps the greatest potential for capturing rich gender data and concepts - conducted sex or gender based analysis at this third level. No articles in our sample employed intersectional analysis (Level 4).

### Data Sources

While the data types and sources utilized by articles in our sample were diverse (table 3), there did not appear to be any particular type of data that corresponded with increased or improved SGBA. Among the qualitative articles, the three that employed a Level 2 approach to SGBA, rather than Level 1, utilized all three data types: legal documents, policy documents, and interviews. The quantitative article that used a Level 2 approach linked clinical trial data with drug adverse effect and medical services data, while the one functioning at the third level was based on survey data. Use of aggregate data that prohibited sex- or gender-disaggregated analysis was common in our sample. The aggregate data phenomenon was especially prominent within the articles employing quantitative methodologies.

**Table 3:** Data sources, by research method of article

Qualitative data sources
Legal and/or policy documents (n = 10)
Focus groups or interviews (n = 4)

Quantitative data sources *

Administrative prescription database (n = 10)
Drug approval data (n = 7)
Survey data (n = 5)
Clinical study/trial data (n = 4)
Drug safety/adverse event data (n = 3)
Other medical services data (n = 2)
Other data source (n = 4)**

* Categories are not mutually exclusive

** Other sources include: drug promotion spending, mathematical models, and various types of drug and drug company information.

## Discussion

### Limitations and Interpretation

Results from this scoping and mapping study are not necessarily representative of all pharmaceutical policy research. MEDLINE is an incomplete index of the world's pharmaceutical policy research, both geographically and in terms of subject coverage. We further limited our scope by including only English-language articles, and only articles from 2008. While this limited date range ensured the most recent complete sample available, the time-limited sample will necessarily reflect trends and events of current interest in 2006-2008. By applying data abstraction and mapping, rather than in-depth qualitative content analysis, our assessment may not reflect nuances of the discourse around sex and gender in the articles we sampled. An in-depth qualitative content analysis of articles in this discipline might shed further light on the nature of inclusion or exclusion of SGBA in this body of research. Likewise, a larger statistically-based study might be able to test for differences among methodological approaches, study countries of origin, or other article attributes, in order to further assess the status of SGBA in the field.

Nonetheless, our results reinforce previous assessments that SGBA implementation has not been terribly successful to date [31,32], even in areas of medicine in which sex and gender have emerged as significant factors [33,34]. They also highlight windows of opportunity for implementing better SGBA in pharmaceutical policy research. In some cases, incorporating SGBA on the third level of the four-approaches model is fairly "low hanging fruit." Examples of article types that may move fairly easily from a sex and gender blind approach to a SGBA approach include qualitative and quantitative studies that draw on interview, focus group or survey data collected for the purpose of the project. Yet, we found no indication that uptake of SGBA was higher among articles utilizing these data types. Similarly, quantitative studies relying on administrative data that presumably contained an individual-level sex field were not particularly likely to conduct sex-stratified analysis.

A minority of pharmaceutical policy issues and research questions do not necessarily tie in with sex and gender issues. Studies distanced from human impacts - e.g., about attributes of published articles, about drug firm behaviours, or about economic incentives for drug development - may be legitimately considered unlikely to have sex- or gender-specific effects that should be examined in the same article. However, studies with human data, studies of human reactions to drugs, and studies of policy impacts on humans can all be reasonably expected to include SGBA. We found many such articles in the Level 1 category lacking SGBA despite discussing a topic known to have sex and/or gender effects, determinants or disproportionate impacts. Thus, it appears that the field of pharmaceutical policy has not fully integrated SGBA guidelines and recommendations into current research practice. In some cases, it would seem that a higher awareness of sex and gender issues among pharmaceutical policy researchers could make such inclusions second nature. However, given that discussions of mainstreaming gender and sex issues into research have been ongoing for many years now, and major research funders offer guidelines on how to conduct such research, it is clear that simple "awareness" is not the only thing lacking.

### Disciplinary Culture Change Opportunities

By failing to consistently apply SGBA in pharmaceutical policy research, we risk incomplete or inaccurate research conclusions about this important component of health care. However, the field of pharmaceutical policy (and health policy studies more generally) might borrow from patient safety literature and frame this as a "systems" deficiency [35]. Rather than blaming individual researchers who are not following SGBA guidelines, perhaps the focus should be placed upon changing the culture of pharmaceutical policy researchers. Such an approach of targeting the culture of researchers, in order to create a social shift, is supported by Rogers' Diffusion of Innovation theory [36], which posits that awareness is merely the beginning stage of acceptance of a new way of functioning. Beyond awareness, individuals must be persuaded before deciding to implement change.

How can pharmaceutical policy researchers be persuaded to implement change? Creating a peer culture that expects to see evidence of SGBA in any paper may provide "checks" for SGBA in the peer review process for papers and presentations. Adoption of SGBA as a routine element in pharmaceutical policy research could be further facilitated by transforming current health research funder guidelines and policies, which may be ignored with little consequence to researchers, into firmer requirements for grant support or renewal. Finally, were journals, or the International Committee of Medical Journal Editors, to require SGBA unless inappropriate, this could significantly impact the frequency with which SGBA appears in the published literature, as acceptability for publication has been identified as a motivating factor for other study attributes [37].

In order to illustrate common approaches to SGBA within contemporary pharmaceutical policy research, we turn our lens inward by critiquing a selection of papers produced by our own team. As a coordinating centre of a national pharmaceutical policy research network, we conduct studies on a wide variety of policy questions using varied methods in our work. The following four selected studies illustrate areas of opportunity for SGBA and, in some cases, areas where SGBA will be acceptably limited. In reflecting on our own research and reporting practices, we find ourselves no less "guilty" of neglecting SGBA than our colleagues: we have failed to either perform or report on sex-disaggregated analyses in quantitative studies, conflated our terminology and failed to look for gendered aspects of policy phenomena.

The first of our papers is an example of a topic for which sex and gender aspects are not apparent, as was the case with a minority of the papers mapped for this study of the literature. This was a study of the correlation between drug pricing policy and research and development (R&D) spending [38]. While there may be definite gendered forces directing R&D expenditures and dictating reactions to pricing policies, the primary focus of the study and the data involved do not provide grounds for *necessarily *conducting or commenting on sex and/or gender themes. Thus, while this article works at Level 1 of the four approaches to SGBA, such an approach is not inappropriate.

The second example illustrates analysis of sex-specific concerns without in-depth SGBA. Published as a medical journal "research letter," the article examined the impact of media reports about drug risks on purchases of a hormonally-based drug by Canadian women [39]. It reported findings of an empirical analysis of a sex-specific concern (potentially dangerous off-label prescribing), but did so without any critical analysis of gender dynamics. Since the article did not reach beyond using women's data to study a women's health issue related to female reproductive capacities, it might be considered a Level 2 approach (biological essentialism). However, we can report from our experience in writing this article (and others for like journals) that medical journal articles have strict word limits and must focus on the (generally clinical) interest of the readership. A Level 4 (intersectional) study of this topic could be done - and would be a valuable contribution to scholarship about gendered dynamics related to medicine use, promotion, risks and harms - but it would not likely be publishable in a general medical journal.

The third example drawn from our work illustrates missed opportunities to conduct meaningful SGBA. In a recent paper published in a health services journal, researchers compared cost-related nonadherence (CRNA) to prescription drugs in the United States and Canada [40]. Findings were adjusted for sex, but analysis was not sex-stratified. While sex differences in CRNA were not the primary outcome of interest, given the mixed evidence on sex or gender as determinants of nonadherence [41], it would have been ideal to have also examined this potential phenomenon in this study. While the study concluded that there is a "Canadian advantage" in prescription adherence, it would have been preferable to know if this advantage is gender and sex neutral or whether it applies more for women or men. Intersecting sex and income (another factor that was adjusted for, rather than stratified in the paper), would go further yet in adding valuable knowledge about CRNA. Although the survey used for this study would not have powered the intersectional ideal, the fact remains that this paper is an example of a study that should have moved beyond a Level 1 approach or, if sample size prohibited, commented on the need to do so in future research.

Power considerations are less likely to be a problem for research with administrative data, as our final example illustrates. In an examination of outpatient prescription drug spending, researchers sex-disaggregated the data but did not explore the interpretation of that analysis [42]. The term "gender" was used throughout the study but the discussion of results reveals that the study conflated terms by using "gender" as a euphemism for "sex." While this paper met our minimal criteria for a Level 3 approach, given that it sex-disaggregated the results, a fuller SBA would have required additional tables of the major finding by sex. Moreover, although the paper explored the effects of sex, income, age, and health status on financial burdens, it was not a Level 4 approach to SGBA because, like most statistical analyses, it assessed the individual contributions of these characteristics to the outcome of interest rather than intersecting them.

## Conclusions

Via a literature mapping method, we investigated the use of sex and gender-related language, inclusion of sex and gender-related concepts, and level of sex and gender based analysis (SGBA) in a sample of contemporary English-language pharmaceutical policy research. We found that the majority of articles could have reasonably been expected to include SGBA but did not. Despite pharmaceutical policy's long entwinement with issues of sex and gender, and the emergence of international guidelines for inclusion of sex and gender in health research, the community of pharmaceutical policy researchers appears not to have internalized, or "mainstreamed," gender and sex analyses. While research funders have adopted SGBA policies, medical journals, which might have greater influence, have not yet followed suit. Increased application of SGBA is, in most cases, not only appropriate for the topics under investigation, but well within the reach of today's pharmaceutical policy researchers. However, this requires consideration of sex and gender factors in research design in order to ensure adequate data sources and analytical frameworks. Fully intersectional analysis, examining the interactions between sex and gender and other social determinants of health, is rarely approached in today's pharmaceutical policy literature. While this is unsurprising, given the relative novelty of intersectional approaches, it should not be seen as prohibitive for pharmaceutical policy research to begin to employ methods that grapple with multiple and overlapping issues of diversity and difference.

## Competing interests

The authors declare that they have no competing interests.

## Authors' contributions

All authors were involved in conceptual development of the project. DG conducted the literature search. DG and AB created the abstracting template and conducted the literature mapping. DG and SM were primarily responsible for writing up the research. All authors approved the final manuscript.

## Appendix: Search Strategy

MEDLINE 1950-present with daily update (Ovid)

13 July, 2009

1. public policy/or health policy/

2. legislation, drug/or "drug and narcotic control"/or drug approval.mp.

3. 1 or 2

4. Pharmaceutical Services/or Insurance, Pharmaceutical Services/or Technology, Pharmaceutical/or Pharmaceutical Preparations/or Pharmaceutical Solutions/

5. (Pharmaceutical$ or Prescription or Prescription Drug$ or Drug$ or Medicine$ or medication$).mp.

6. 4 or 5

7. 3 and 6

8. limit 7 to yr="2008"

9. limit 8 to english language
